# Blood DNA methylation pattern is altered in mesial temporal lobe epilepsy

**DOI:** 10.1038/srep43810

**Published:** 2017-03-09

**Authors:** Hong-Yu Long, Li Feng, Jin Kang, Zhao-Hui Luo, Wen-Biao Xiao, Li-Li Long, Xiao-Xin Yan, Luo Zhou, Bo Xiao

**Affiliations:** 1 Department of Neurology, Xiangya Hospital, Central South University, Changsha, Hunan 410008, China; 2 Department of Anatomy and Neurobiology, Central South University School of Basic Medicine, Changsha, Hunan 410013, China; 3 Key Laboratory of Hunan Province in Neurodegenerative Disorders, Changsha, Hunan 410008, China

## Abstract

Mesial temporal lobe epilepsy (MTLE) is a common epileptic disorder; little is known whether it is associated with peripheral epigenetic changes. Here we compared blood whole genomic DNA methylation pattern in MTLE patients (n = 30) relative to controls (n = 30) with the Human Methylation 450 K BeadChip assay, and explored genes and pathways that were differentially methylated using bioinformatics profiling. The MTLE and control groups showed significantly different (P < 1.03e-07) DNA methylation at 216 sites, with 164 sites involved hyper- and 52 sites hypo- methylation. Two hyper- and 32 hypo-methylated sites were associated with promoters, while 87 hyper- and 43 hypo-methylated sites corresponded to coding regions. The differentially methylated genes were largely related to pathways predicted to participate in anion binding, oxidoreductant activity, growth regulation, skeletal development and drug metabolism, with the most distinct ones included SLC34A2, CLCN6, CLCA4, CYP3A43, CYP3A4 and CYP2C9. Among the MTLE patients, panels of genes also appeared to be differentially methylated relative to disease duration, resistance to anti-epileptics and MRI alterations of hippocampal sclerosis. The peripheral epigenetic changes observed in MTLE could be involved in certain disease-related modulations and warrant further translational investigations.

Temporal lobe epilepsy (TLE) is a common neurological disease that may affect up to 1% population, representing a significant healthcare and financial burden to society and families^1^,^2^. Epidemiological studies estimate an approximate 0.5% morbidity of TLE in China (9 million patients nationwide), with 5–6 million patients requiring continuous medical attention and 0.4 million new patients being diagnosed each year. Youth and adults are mostly affected, with a higher prevalence in rural than urban areas^3^,^4^. Mesial temporal lobe epilepsy (MTLE) is the most common type of TLE, featured by refractory seizures worsen with time, and often become resistant to anti-epileptic therapies and require brain surgery to control symptoms^5^,^6^. The clinicopathological manifestations of MTLE appear to be largely related to lesions of the medial temporal lobe structures^7^,^8^,^9^. Atrophy or sclerosis of the hippocampal formation (HS) and neighboring limbic structures can be detected via brain imaging, with neuronal death, granule cell dispersion, mossy fiber sprouting, neuronal network reorganization, gliosis and inflammation found histologically in affected brain regions^10^,^11^,^12^,^13^,^14^,^15^,^16^,^17^,^18^,^19^,^20^,^21^.

While great progress has been made in the past decades in understanding of the neuropathology and pathophysiology of MTLE, the etiological and pathogenic underpinnings of this disorder remain poorly understood^5^,^6^,^19^,^20^,^21^. Biochemical and bioinformatics approaches are used in recent years to study MTLE, revealing broad changes in protein, mRNA and DNA expression and/or modulation in the brain^22^,^23^,^24^. For instance, proteomic studies have shown remarkable and dynamic changes in protein expression involving multiple cellular systems in epileptic brain^25^,^26^,^27^,^28^,^29^,^30^. Transcriptomic studies indicate that a few hundred genes are either up- or down-regulated in the ictogenic relative to unaffected brain regions^31^,^32^,^33^,^34^,^35^,^36^,^37^. Epigenetic changes are being recognized as a part of the molecular reconfiguration in TLE, with a wide array of genes involved in neuronal/synaptic transmission, cell survival/death and transcriptional regulation differentially methylated in the brains of patients^38^,^39^,^40^,^41^,^42^.

Recent epigenetics studies have shown blood DNA methylation changes in a number of neurological and psychiatric diseases, including Alzheimer’s disease, Parkinson’s disease, Down’s syndrome, infantile spasm, depressive disorders and schizophrenia^43^,^44^,^45^,^46^,^47^,^48^,^49^,^50^,^51^,^52^,^53^,^54^,^55^,^56^. Investigations into peripheral DNA methylation changes are considered important to help develop novel biomarkers for early diagnosis of some brain diseases. Here we carried out a case-control study on blood whole-genome DNA methylation pattern in 30 MTLE patients relative to sex/age-matched controls, and have identified a panel of differentially methylated genes involved in multiple interactive molecular pathways between the two groups.

## Results

### Sample and assay quality controls

The integrity of purified blood DNA was evaluated with agarose gel electrophoresis. In ethidium bromide stained gels, the bulk of DNA was migrated at locations relative to molecular sizes apparently greater than 15000 base-pairs (bp), arranged largely as two distinct bands. The pattern of DNA bands was comparable across all samples, without smearing of labeling signal (Supplemental Fig. 1). Several sample-independent assay controls were carried out, which verified the efficacy and specificity of the Beadchip. (1) Staining controls indicated that the signal of dinitrophenyl (DNP) (green fluorescent channel) and biotin (green fluorescent channel) attached beads was sufficient; (2) Hybridization controls indicated that the signal (green fluorescence) was intensified with the increase of biotin-tagged fluorescent probes in a concentration-dependent manner; (3) Target removal controls revealed a loss of fluorescent signal in conditions whereby the oligos were extended using the probe sequence as template; (4) Extension controls showed that the signal was enhanced with the extension of either the guanine-cytosine (GC) residues (green fluorescence) or adenine-thymine (AT) residues (red fluorescence) (Supplementary Fig. 2).

Sample-dependent assay controls were used to verify the efficacy of bisulfite sodium conversion on the Illumina 450K Beadchip system. In Infmium-I assay controls, the hybridization signal appeared properly strong as tested with three quality control probes (C1–3) for the green fluorescence (Supplementary Fig. 3A) and three control probes (C4-6) for the red fluorescence (Supplementary Fig. 3B). In Infmium-II assay control, four probes (red fluorescent) were used to verify the efficacy of bisulfite sodium conversion involving the cytosine residues, which yielded strong red fluorescence (Supplementary Fig. 3C,D).

### Overall changes in blood genomic DNA methylation in MTLE

**Figure 1:**
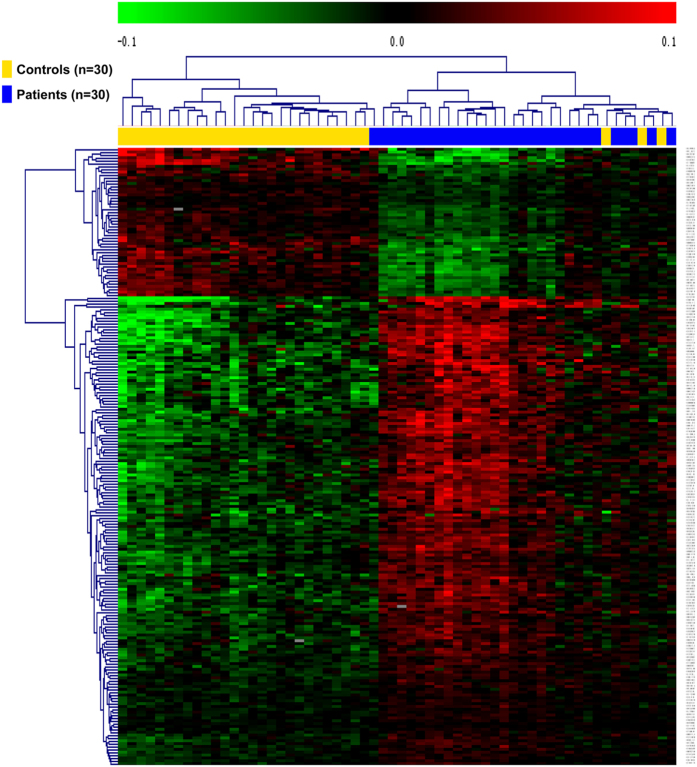
Heat map generated from clustering-analysis of microarray data illustrating differentially methylated DNA sites in blood whole genome in the mesial temporal lobe epilepsy (MTLE) patient group (n = 30) relative to the control group (n = 30) as indicated above the heat map. Bead tags are listed vertically at the right side of the map. The extents of green and red represent levels of hyper-methylation, hypo-methylation, respectively, whereas black indicates no change in methylation, relative to control.

**Figure 2:**
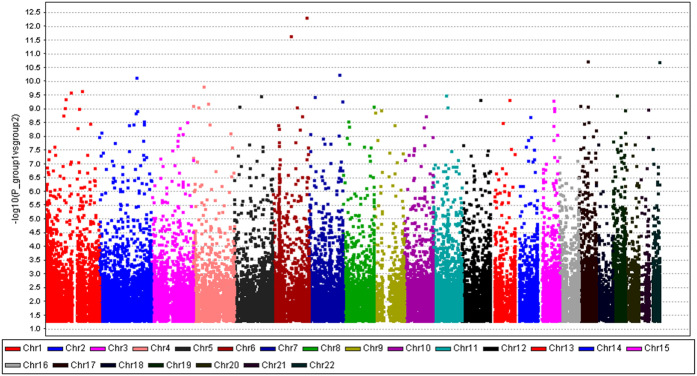
Manhattan scatter diagram illustrating the distribution of differentially methylated DNA sites in the human genome relative to individual chromosomes. The differentially methylated sites, including the 216 sites with P < 1.03e-07 by Bonferroni correction test, appear to distribute across all 22 chromosomes without apparent preference.

Using the Genome studio V2011 software, the β-values of 485,577 DNA methylation sites were reported for the samples from individual control (n = 30) and patient (n = 30) subjects (Fig. 1). Statistical analysis indicated that a total of 216 sites showed difference in the degree of methylation between the two groups (defined using a cutoff P value < 1.03e-07). Among these differentially methylated sites, 164 sites involved hyper-methylation, whereas 52 sites were hypo-methylated, yielding a ratio of 3.15 (Supplementary Table 1). These differentially methylated sites appeared to distribute on the 22 chromosomes without an apparent preference (Fig. 2).

**Figure 3:**
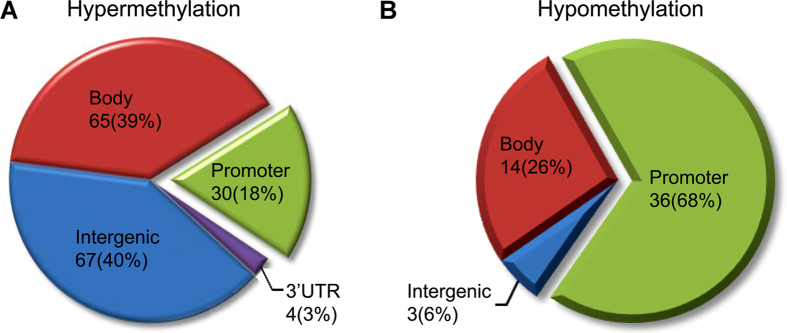
Pie graphs illustrating the rate distribution of differentially methylated DNA sites in mesial temporal lobe epilepsy in reference to genomic structure domains. Percentage rates for the hyper-methylated and hypo-methylated sites associated with the promoter, coding (body), non-coding (intergenic) and the 3′ UTR regions are illustrated in (**A**) and (**B**), respectively.

Further analysis in reference to DNA functional domains, among the hyper-methylated sites there were 67 sites located at non-coding regions (67%), 65 sites at coding domains (39%), 30 sites associated with promoters (18%) and 4 involved the 3′ UTR terminal (3%). Among the hypo-methylated sites, there were 3 sites occurred in non-coding intergenic regions (6%), 14 sites at coding domains (26%), 36 sites associated with promoters (68%), and none related to the 3′ UTR end (Fig. 3).

**Figure 4:**
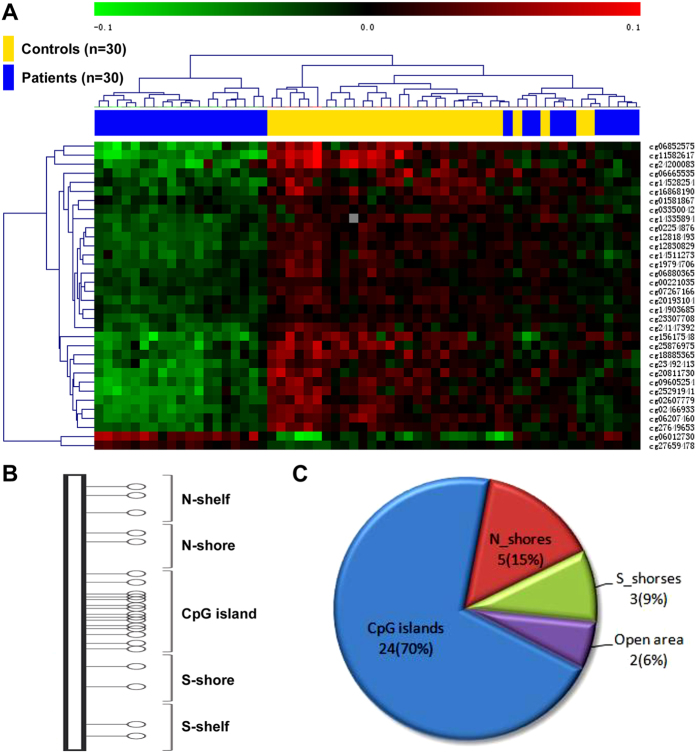
Analysis of differentially methylated DNA sites around the CpG island structures in mesial temporal lobe epilepsy. Panel (A) illustrates the heat map derived from clustering analysis of the microarray data from individual control (n = 30) and patient (n = 30) subjects as indicated above the heat map. Panel (B) shows the organization of the CpG island structures. Panel (C) plots the percentage distribution of the differentially methylated DNA sites at corresponding CpG island domains.

Among the 66 differentially methylated sites over promoter regions (Fig. 4A), the ratio of hyper- to hypo-methylated sites was 0.83 (30/36), which was lower than the overall ratio (164/52 = 3.15) of the entire set of hyper- to hypo-methylated DNA sites in the whole genome. A total of 36 differentially methylated sites were associated with the CpG island structures (Fig. 4B), mostly involved hypo-methylation (34/36). Thus, the 34 hypo-methylated sites occurred largely at the CpG islands per se (24), with the remainder at the N-shores (5), S-shores (3) and Open area (2), whereas none in the N- and S-shelves (Fig. 4C).

### Cross-validation of methylation with pyrosequencing

**Figure 5:**
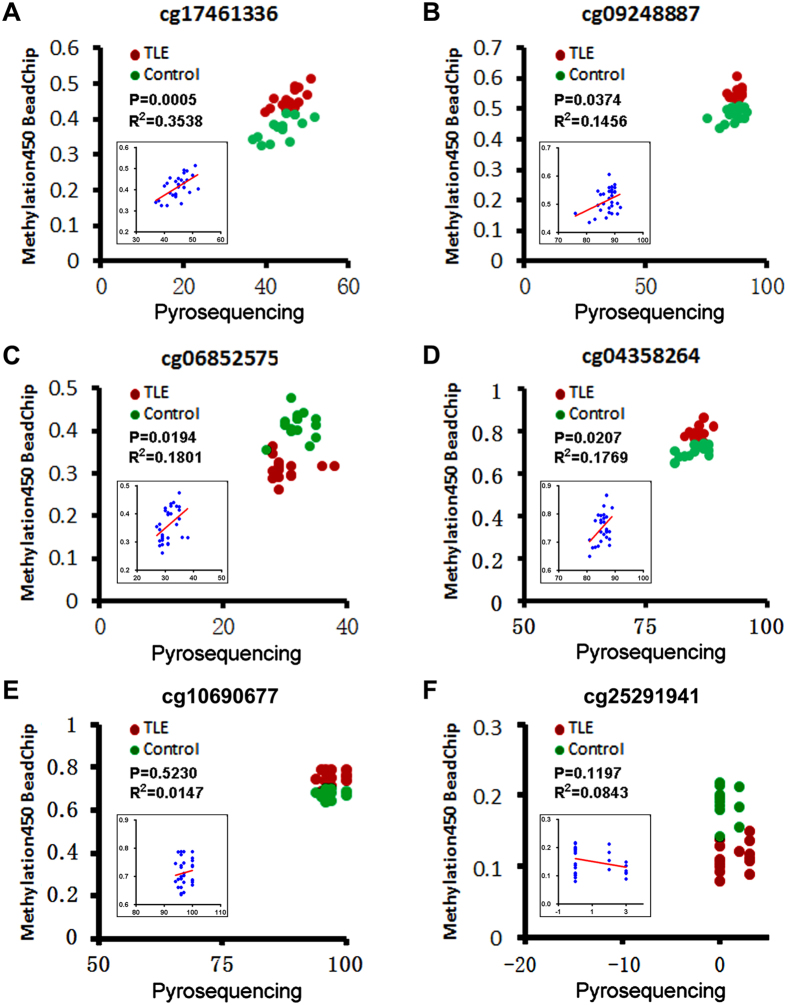
Cross-validation of DNA methylation with the pyrosequencing. Shown are degrees pf methylation of six representative CpG loci reported by Methylation450 BeadChip (Y axis, ratio) and pyrosequencing (X axis, percentage) assays. Red and green dots in each panel plot values of methylation at a given site among individual patients (n = 15) with temporal lobe epilepsy (TLE) and controls (n = 15). Insert in the panel shows a correlation analysis between the two sets of values among individual cases regardless of subject groups, with P and R^2^ values labeled for each gene site. For cg17461336 (**A**), cg09248887 (**B**), cg06852575 (**C**) and cg04358264 (**D**), the degrees of methylation detected by the two methods were positively correlated (P < 0.05) in reference to individual samples. The two methods consistently reveal an exceptionally high methylation of cg10690677 (**E**) and minimal methylation of cg25291941 (**F**) among individual subjects, while there is no correlation between the two sets of values for either site in a case-based manner (likely due to ceiling and floor effects, respectively).

In order to evaluate the accuracy of DNA methylation detected with the Methylation450 BeadChip assay, a subset of CpG loci with varying degree of methylation was selected for additional methylation analysis using the pyrosequencing method. Thus, patient (n = 15) and control (n = 15) blood samples were subjected to methylation analysis at eight loci (cg17461336, cg09248887, cg06852575, cg04358264, cg10690677, cg21593669, cg25291941, cg11582617) via PCR amplification followed by pyrosequencing. Pyrosequencing revealed methylation of cg17461336 at 45.7 ± 3.1% in the MTLE group and 43.8 ± 4.1% in the control group (Fig. 5A), which was correlated with the data from Methylation450 BeadChip test when analyzed against individually samples (P = 0.0005, R^2^ = 0.3548) (Fig. 5A, insert). The cg09248887 site had methylation rate at 88.2 ± 1.9% in the MTLE group and 86.6 ± 4.3% in the control group by pyrosequencing (Fig. 5B), significantly correlated with the BeadChip data relative to individual subjects (P = 0.0374, R^2^ = 0.1456) (Fig. 5B, insert). For cg06852575, the methylation rates were 30.2 ± 2.9% in the MTLE group and 32.0 ± 2.2% in the control group (Fig. 5C), consistent with the BeadChip data referring to individual cases (P = 0.0194, R^2^ = 0.1801) (Fig. 5C, insert). For cg04358264, the methylation rates were 85.9 ± 1.4% in the MTLE group and 85.3 ± 2.5% in the control group (Fig. 5D), also in line with the BeadChip data among individuals (P = 0.0207, R^2^ = 1769) (Fig. 5D, insert). The cg10690677 (Fig. 5E) and cg21593669 (not shown) sites showed very high (near 100%) methylation among patient and control samples with both methods, although there was no correlation between the two sets of value as analyzed against individual cases (likely due to a ceiling effect) (Fig. 5E, insert). Opposite to the above, the loci cg25291941 (Fig. 5F) and cg11582617 (not shown) had very low methylation (near 0%) by either method, and there was also no correlation between the two sets of data among individuals (likely a floor effect) (Fig. 5F, insert).

### Relevance of differentially methylated DNA sites to functional genes

The differentially methylated sites revealed by the BeadChip assay were analyzed in relevance to known functional genes against the DAVID bioinformatics database (http://david.abcc.ncifcrf.gov/home.jsp/). Among the 216 differentially methylated sites, a sum of 130 sites was associated with known functional genes, with 87 sites being hyper-methylated, whereas 43 sites hypo-methylated. A subset of genes in either the hyper- or hypo-methylated genes was shown to have single nucleotide polymorphism (SNP) from query sites (Supplementary Table 1). The top ten genes with the greatest extent of hyper-methylation were ZNF638, CYP3A43, UNC80, AIRE, RBPJL, SCGB1D1, FAM49B, FLJ44606, PYY and NLK. The hyper-methylated loci were at the body region among eight of the above ten genes, and three of them had SNPs (Table 1). The top ten genes with the greatest extent of hypo-methylation were SERAC1, ADCY5, HOXD11, ZNF257, CLTCL1, ZNF702P, GORASP2, POP1 and TSPAN3. Among this latter set, the hypo-methylated sites occurred largely (n = 7) around the promoter region, while none was associated with SNP (Table 1).

### Ontological profiling of differentially methylated blood genes in MTLE

With bioinformatics analysis per the DAVID and GO databases, the 216 differential DNA methylation sites could be linked to multiple genes arranged in 4 groups in context of cellular component (CC) pathways. These included (1) GO:0030529~ribonucleoprotein complex (involving 8 differentially methylated genes, P = 0.077 between MTLE and control groups by *t*-test, same data presenting format below); (2) GO:0005792~microsome (5 genes, P = 0.092); (3) GO:0031981~nuclear lumen (16 genes, P = 0.094) and (4) GO:0042598~vesicular fraction (5 genes, P = 0.094). Statistical analyses indicated that the extent of methylation appeared to approach significant differences between the MTLE and control groups for each of the above CC systems, with the fold of enrichment ranging from 1.5 to 3 (Supplementary Table 2).

The differentially methylation sites could be linked to 9 groups in context of biological processing systems (BP pathways). These included (1) GO:0040008~regulation of growth (involving 8 differentially methylated genes, P = 0.010); (2) GO:0001501~skeletal system development (7 genes, P = 0.025); (3) GO:0048705~skeletal system morphogenesis (4 genes, P = 0.044); (4) the GO:0016055~Wnt receptor signaling pathway (4 genes, P = 0.067); (5) GO:0009952~anterior/posterior pattern formation (4 genes, P = 0.076); (6) GO:0009123~nucleoside monophosphate metabolic process (3 genes, P = 0.078); (7) GO:0010720~positive regulation of cell development (3 genes, P = 0.085); (8) GO:0006164~purine nucleotide biosynthetic process (4 genes, P = 0.086) and (9) GO:0006396~RNA processing (8 genes, P = 0.090). The extent of methylation changes showed statistically significant differences for groups (1), (2) and (3) between the MTLE and control groups, with the fold of enrichment ranged from 2 to 6 (Supplementary Table 3). Multiple genes participated in the above significantly altered BP pathways, including TMX1, MAPT, PPP2CA, MAEL, NPR1, WRN, E4F1 and AFG3L2 in group (1); TAPT1, ALOX15, HOXB2, ANKRD11, DLL3, COL11A2 and HOXD11 in group (2); and HOXB2, ANKRD11, COL11A2 and HOXD11 in group (3).

In context of molecular function (e.g., biochemical cascade), the differentially methylated genes were organized into 11 groups, including (1) GO:0043168~anion binding (5 genes, P = 0.006); (2) GO:0043167~ion binding (44 genes, P = 0.019); (3) GO:0031404~chloride ion binding (4 genes, P = 0.022); (4) GO:0016712~oxidoreductase activity (3 genes, P = 0.022); (5) GO:0004886~retinoid-X receptor activity (2 genes, P = 0.067); (6) GO:0046872~metal ion binding (40 genes, P = 0.079); (7) GO:0030375~thyroid hormone receptor coactivator activity (2 genes, P = 0.082); (8) GO:0010861~thyroid hormone receptor activator activity (2 genes, P = 0.082); (9) GO:0003708~retinoic acid receptor activity (2 genes, P = 0.089); (10) GO:0043169~cation binding (40 genes, P = 0.089) and (11) GO:0005506~iron ion binding (6 genes, P = 0.089). The extent of differential methylation showed significant differences between the patients and controls for groups (1) to (4), including genes such as GABRB1, SLC34A2, CLCN6, CLCA4, CYP3A43, CYP3A4, CYP2C9 that are critically involved in ion channel functions and oxidoreductant activity. The fold of enrichment varied greatly from over 1 to nearly 30 (Supplementary Table 4).

### Genomic network profiling of differential methylated blood genes in MTLE

**Figure 6:**
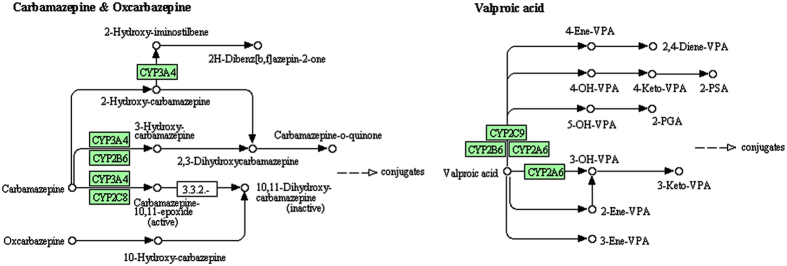
Informatics analysis showing an example of the differentially methylated genes as a part of the hsa00980 pathway. This pathway participates in the metabolism of multiple drugs (see Supplementary Fig. 4). Shown are the parts related to the metabolism of several commonly used antiepileptics, i.e., carbamazepine, oxcarbazepine and valproic acid. Images are obtained from KEGG (http://www.kegg.jp/kegg/kegg1.html) with permission (Ref. #: 16835).

With bioinformatics assessment against the KEGG database (http://www.genome.jp/kegg/), a total of 6 genomic networks/pathways were generated in context of potential biological interplays between/among the genes with differential methylation in MTLE (Table 2). These pathways were consolidated as (1) hsa00591: Linoleic acid metabolism; (2) hsa00983: Drug metabolism. (3) hsa00830: Retinol metabolism; (4) hsa00980: Metabolism of xenobiotics by cytochrome P450; (5) hsa00982: Drug metabolism; and (6) hsa00140:Steroid hormone biosynthesis. Statistically, the extent of differential methylation was significantly different between the patient and control groups for pathways (1) through (5), but not for pathway (6), with the differentially methylated pathways (hsa00980, hsa00983, hsa00982) involved in drug metabolism. The fold of enrichment among these sets of pathways ranged from over 7 to more than 16 (Table 2). Further bioinformatics analysis indicated that the hsa00980 pathway plays a critical role in drug metabolism (Supplementary Fig. 3), especially for the processing of the commonly used antiepileptics in the clinic, including carbamazepine, oxcarbazepine and valproic acid (Fig. 6).

### Preliminary analysis of differential DNA methylation relative to disease course, anti-epileptics resistance and MRI abnormality

**Figure 7:**
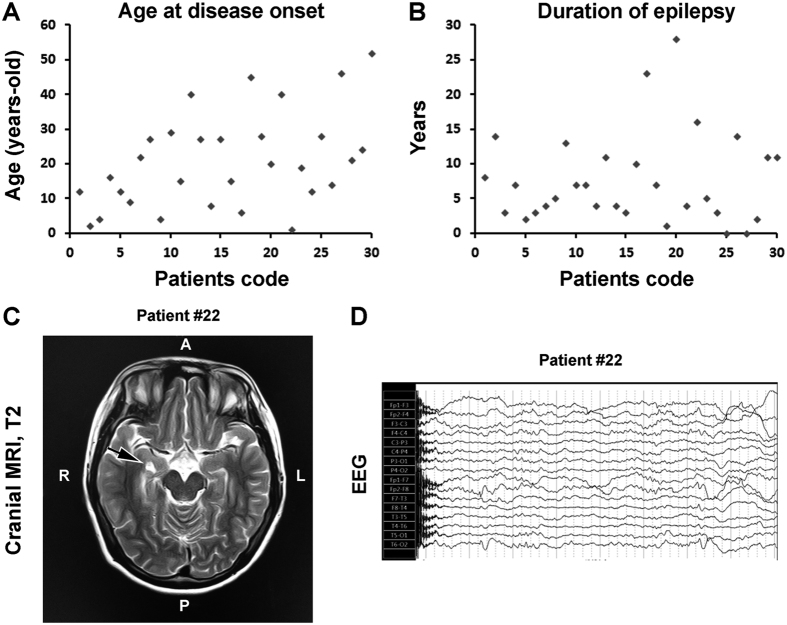
Case-sensitive presentation of age at onset and duration of epilepsy, and representative brain imaging and electroencephalograph (EEG) script from the patient group. Dot graphs plot the age of onset (**A**) and duration (**B**) of epilepsy among the 30 patients listed from 1–30. Panel (C) shows a cranial T2 weighted Magnetic Resonance Imaging (MRI) film from patient #22, with the arrow pointing to enhanced T2 signal suggestive of sclerosis of the right-side hippocampus. Panel (D) shows slow and spike waves recorded on regions representing the right temporal lobe of this patient during awake and sleep, especially evident at anterior temporal area.

The patient group available for the present study was not large; we carried out preliminary analyses on DNA methylation relative to some diseases sub-characteristics (Table 3; Fig. 7). Two-third cases of the group had a disease course for 10 years and longer; we found 9 genes in these patients with significantly differential methylation relative to the cases with disease duration less than 10 years, including the solute carrier family 45 member 4 and low density lipoprotein adaptor protein 1. Among the top 10 hypermethylated and top 10 hypomethylated DNA loci, each involved 9 genes (Supplementary Table 5). The extent of methylation was different at five functional genes between patients that were clinically assessed as being anti-epileptics resistant and sensitive, including the microtubule associated protein 1 light chain 3b, active BCR-related, kallikrein peptidase 14, allantoises and MAGI family member. Except for the first gene, this group represented the known gene-coding sites of the top 10 hypermethylated loci. Among the 10 hypomethylated loci, nine of which involved DNA coding areas (Supplementary Table 6). MRI abnormality was reported among a half of the patients, while 9 cases of them had hippocampal sclerosis (Table 3; Fig. 7C). Give that hippocampal sclerosis is pathologically characteristic of MTLE, we analyzed methylation profile between patients with hippocampal sclerosis relative to those lacked MRI detected brain abnormality (n = 14) (Table 3). In the top 10 differentially methylated sites, there were 7 gene-coding DNA loci, of which 3 were among the most hyper-methylated genes, while 4 belonged to the most hypo-methylated genes, respectively (Supplementary Table 7). While we attempted to profile the above differential methylation against the DAVID, GO and KEGG databases, no specifically remarkable associations were found between disease course, anti-epileptics resistance or MRI abnormality with particular GO pathways or genomic networks (data not shown).

## Discussion

DNA methylation serves an important epigenetic mechanism for regulation of gene expression. It may occur at the coding, promoter or other regions of genomic sequence in a tissue and cell specific manner, and might be significantly altered in disease conditions^57^. In particular, methylation of the CpG island regions, which is often associated with promoters in mammalian genome, may play a key role in controlling the expression of corresponding downstream genes^58^. Although the relationship between peripheral epigenetic profile and central nervous system conditions could be very complex, exploring peripheral epigenetic alterations in brain disorders is considered a direction with translational significance, given that in most clinical settings only peripheral samples, especially blood, are available. Differential central and peripheral DNA methylation is reported lately in a variety of neurological and psychiatric disorders, with some studies showing a parallel pattern change in brain and blood derived samples^43^,^44^,^45^,^46^,^47^,^48^,^49^,^51^,^52^,^53^,^54^,^55^,^56^. For TLE, emerging data point to a potential role for aberrant epigenetic modulation in its etiology, pathogenesis or ictal pathophysiology^37^,^38^,^39^,^40^,^41^,^42^,^59^,^60^, while less is known about the peripheral epigenetic change in this disorder.

The present study extends a first set of human data demonstrating differential DNA methylation in blood genome in MTLE. We have identified 216 differential DNA methylation sites, with 164 sites being significantly hyper-methylated and 52 sites hypo-methylated, in the MTLE patient relative to healthy control groups, according to Methylation450 BeadChip analysis. We cross-validated DNA methylation with the pyrosequencing method at a subset (8) of CpG loci. Overall, methylation data reported by the two methods are consistent among this set of loci that have varying degrees of methylation. In fact, the methylation data reported by BeadChip and the pyrosequencing methods are positively correlated in a case-based manner (regardless of experimental groups) for four sites.

The Beadchip assay system can theoretically report methylation changes at 485,577 sites across the whole genome. Thus, the differential DNA methylation appears to occur at a fairly low rate (i.e., 4/10000) in relative to the whole blood genome (using a cutoff P value < 1.03e-07 for multi-factorial statistical testing). De- or hypo-methylation in the promoter/CpG island regions is generally considered to enhance the expression of downstream genes^50^,^58^. In this study, the overall ratio of hyper- to hypo-methylated sites around the promoter regions was 0.83, with that for the CpG islands and surrounding structures even lower (2/34 or 0.0625), suggestive of a predominantly down-regulated methylation at these structures. One may expect that such changes could enhance the expression of some corresponding genes. Of note, the incidence of methylation at the promoter regions as assessed in brain samples has been shown to be increased (about 81.5%) in patients with MTLE^42^, but reduced (over 90%, assessed in hippocampal tissue) in a mouse model of TLE^60^.

The bioinformatics profile of our data indicates that the differentially methylated regions can be correlated to 130 known functional genes. Among the top ten hyper- and top ten hypo-methylated genes (Table 2), a subset (i.e., GABRB1, LC34A2, CLCN6, CLCA4, CYP3A43, CYP3A4, CYP2C9) has been related to epilepsy in epidemiological, pathophysiological or clinical context (to be discussed further). Thus, our current data suggest that DNA methylation changes in MTLE can occur in many genes that are not (yet) known to be associated with epilepsy or seizure disorders. This finding is in line with the notion that multiple genetic/epigenetic factors could be involved in TLE or seizure disorders^42^.

Ion channel dysfunction represents a fundamental pathophysiological element underlying abnormal neuronal discharge in seizure disorders, with evidence supporting an involvement of genetic and epigenetic factors in epileptic channelopathies^37^,^39^,^61^,^62^. Mutations of the genes (SCN1A, SCN1B, SCN2A) coding some sodium channel subunits cause enhanced excitability of glutamatergic neurons and/or reduced excitability of GABAergic neurons in familial seizure disorders^63^,^64^,^65^,^66^,^67^,^68^. Mutation of the gene coding the chloride channel protein (CLCN2) might also potentiate neuronal excitability in idiopathic epilepsy^69^,^70^. In the present study, genes coding the chloride channel 6 (CLCN6), chloride channel accessory 4 (CLCA4), sodium carrier (SLC34A2) and GABA receptor subunit (GABRB1) proteins show differential methylation in the MTLE relative to control groups, based on blood sample testing.

Resistance to antiepileptic drugs in MTLE can lead to progressive neuropathology and poor prognosis among many patients. Although the underlying mechanism remains unclear, changes in the enzyme systems responsible for drug metabolism could be a modulatory factor. The cytochrome P450 superfamily of proteins (CYPs) plays a major role in drug metabolism, accounting for about 75% of the total metabolism, especially deactivation and clearance of many drugs^71^,^72^,^73^. In the present study, several genes coding the members of CYPs, including CYP3A4, CYP3A43 and CYP2C9, are among the top ten differentially methylated genes in the MTLE relative to control groups. Our GO and KEGG bioinformatics analyses further indicate that they are part of the drug metabolic pathways responsible for some commonly used antiepileptic drugs, such as carbamazepine, oxcarbazepine and valproic acid. It should be noted that the epigenome-wide association analysis in a recent study with brain samples from TLE patients shows no significant methylation changes of the CYPs^42^. Therefore, the finding of differential methylation in CYP3A43, CYP3A4 and CYP2C9 in blood DNA samples in the present study appears to more likely reflect a peripheral epigenetic modulation.

The current study also reveals differential DNA methylation at genomic loci related to some other biological, molecular or signaling systems. Specifically, the genes or genomic interplaying pathways involved in cell/tissue morphogenesis and development, steroid hormone biosynthesis and metabolism as well as skeletal system development exhibit significantly differential methylation in the current MTLE relative to control groups. Further studies will be needed to understand these epigenetic changes in biological and clinical perspectives. Nonetheless, it is noteworthy that many clinical studies have suggested that chronic epilepsy or perhaps the long-term use of antiepileptic drugs may cause growth/developmental problems in children, while abnormal steroid hormone metabolism and certain forms of osteopathy are noted in patients under drug treatments^74^,^75^,^76^,^77^,^78^. In the current cohort of male and female patients and controls, the mean ages were 25 to 35 years, while one-third of the patients with epilepsy onset before 12 year-old (Table 3).

TLE in general and MTLE in specific are complex in clinical setting, and may present with a great spectrum of neurological symptoms, location/extent of cerebral lesions in imaging study, pattern of EEG alterations. Upon receiving medical attention, they are treated with anti-epileptics and other drugs accordingly. This was the case for the epileptic patients enrolled in our current study. While we have attempted to profile the DNA methylation data from the patient group relative to demographic, clinical and brain imaging features using bioinformatics tools, the results do not yet allow a clear conclusion whether specific blood DNA methylation changes are related to several subtype clinical features (disease duration, anti-epileptics resistance and hippocampal sclerosis). Thus, studies with larger patient cohort would be needed for stratified analysis to determine if unique differential blood DNA methylation can be linked to particular subclinical features of MTLE, therefore allowing further development as peripheral biomarkers of this disorder.

## Conclusion

In summary, the present study presents a preliminary set of human data indicating differential DNA methylation at multiple loci of whole blood genome in mesial temporal lobe epilepsy relative to healthy control. Based on bioinformatics analysis, some of the differentially methylated sites are associated with promoters, genes and molecular pathways important for biological and metabolic regulation of anion binding/ion channel activity, oxidoreductant activity, drug metabolism, growth regulation and skeletal system development. These epigenetic changes might reflect some of the pathogenic and/or compensative biological modulations in mesial temporal lobe epilepsy or epilepsy/seizure disorders in general.

## Methods

### Subjects

Patients and controls were enrolled into the current study following written informed consent obtained from all subjects. All research protocols were approved by the Ethics Committee of Central South University, Xiangya School of Medicine and the affiliated Xiangya Hospital, with experiments carried out in accordance with the guideline for study involving human subjects. A total of 30 patients (18 males and 12 females) and 30 sex- and age-matched healthy controls were recruited through our epilepsy clinic from October, 2010 to October, 2014. Patients and controls were all Han Chinese, without previous history of seizure disorders or familial history of other neurological diseases (excluding secondary seizure disorders). All patients met the criteria for the diagnosis of MTLE set by the International League Against Epilepsy (ILAE), including clinical characteristics of simple or complex partial seizures and ictal scalp electroencephalogram (EEG) findings of spike, sharp, slow-spike and slow-sharp waves related to medial temporal lobe regions. Abnormal brain magnetic resonance image (MRI) findings, including hippocampal sclerosis, encephalomal acia foci, lacunar infarction or encephalomal atrophy, were present in approximately a half of the patients. Patients were treated with common anti-epileptics including carbamazepine, oxcarbazepine and valproic acid to control symptoms according to an individually-managed principle. The demographic, clinical, brain imaging and EEG profiles of the subjects are summarized as Table 3, with some metric data (age at disease onset and duration of epilepsy) and representative MRI and EEG presented as Fig. 7.

### Whole blood DNA extraction

Frozen blood samples from patients and control subjects were thawed at 37 °C, followed immediately by whole blood DNA extraction using a commercial kit (Beijing Adly biological company, Beijing, China) according to manufacturer’s instructions. DNA concentration and purity were determined in a NanoDrop spectrophotometer by measuring the optic density ratio of maximal absorbent wavelengths at 260 nm to 280 nm (OD260/280) using salmon sperm DNA as an internal standard. Samples with DNA concentration greater than 50 ng/μl and OD260/280 ratio from 1.60 to 2.10 were used for further experimentations. Integrity of the extracted DNA from all samples was checked by agarose gel electrophoresis with ethidium bromide stain.

### BeadChip DNA methylation assay

The pattern and extent of DNA methylation were assayed using the Infinium HumanMethylation450 BeadChip Kit (Illumina, Inc., San Diego, CA, USA). The kit contained 12332 probes, reporting sample-dependent and independent DNA methylation at specific genomic sites or for assay quality control. DNA samples were processed via a series of steps, including DNA denaturation, whole genome amplification, fragmentation, precipitation, resuspension and hybridization, according to manufacturer’s protocols. Signal (grey scale) of the final hybridization products was captured with the iScan system (Illumina, Inc.). The extent of methylation at a given site was determined by the β value, calculated as β = max (signal B, 0)/[max (signal A, 0) + max (signal B, 0) + 100], ranging from 0–1, representing completely not to totally methylated.

### Pyrosequencing characterization of selected DNA methylation loci

After obtained information about the differentially methylated sites from the Methylation450 BeadChip analysis, we selected eight CpG loci with varying extent of methylation (cg17461336, cg09248887, cg06852575, cg04358264, cg10690677, cg21593669, cg25291941 and cg11582617) for evaluation by means of Pyrosequencing, as a measure of assay cross-validation. To this end, blood DNA samples from 15 MTEL cases and 15 sex and age-matched control cases were bisulfited, followed by PCR amplification of the regions of interest using the PyroMark PCR kit (Qiagen, CA, USA) according manufacturer’s instruction. Nucleotide probes included a biotinylated version allowing detection by streptavidin sepharose, as listed below: (1) cg17461336: GGTTAATTAGGGTATGATATAGAGTAAGA (F), ACCCAAATATACACCACCTAAAT (R-bio) and GGGTATGATATAGAGTAAGATT (S); (2) cg09248887: TGAGGTTATTGTTAGGGAATAGGAGAT (F), CCTTCATCAATCATCCTCCCTTCAAA (R-bio) and CACAATACTTAATTTTTTAACTTC (S); (3) cg06852575: GATTTGTAGGTATTGGGAGATTTT (F), CAAATTCCAACCACCCCCTTC (R-bio) and AGAAATGGTGAGAGTG (S); (4) cg04358264: AATGGGTAAAGTTATAGTGGATTAATT (F), AACCACTTTACAAAAATATTATCAAATACT (R-bio) and AATTAAATTTTAGTTTTGATAAAGG (S); (5) cg10690677: AGTTGATTGTTTTAAAAGGGTAAAGG (F), AACAAAAATCCTCTATTCCAACAAACTATC (R-bio) and GGTGGAGTAGGTTGAGTAGA (S); (6) cg21593669: AGATTGTAGGAGTAGGAGGTATAGTATTA (F), AACCAAAAAACTAAACAACATCTCTAC (R-bio) and GGAGGTATAGTATTAGGATG (S); (7) cg25291941: TGAAGGGGTTGTAAGAGGG (F), ACTCCCAACTCTCTATAAATCTT (R-bio) and TGTAAGAGGGAAGGT (S); and (8) cg11582617: GGAAGTTATTGTGGGTGGAGTTAG (F), ACCCCCTTATCTAAAAAACCCTACTCTT (R-bio) and GTGGGTGGAGTTAGT (S). Pyrosequencing assay was carried out on a PyroMark Q24 instrument using the PyroMark Gold Q24 Reagents (Qiagen, CA, USA). Purification and subsequent processing of the biotinylated single-stranded DNA were performed following the manufacturer’s recommendations.

### Data and bioinformatics analyses

Differential DNA methylation sites were selected and analyzed with the BeadStudio Methylation Module v3.2 software (Illumina, Inc.) (http://www.illumina.com). The Pyrosequencing data were analyzed using the PyroMark Q24 software (Qiagen, CA, USA). R language (https://www.r-project.org/) was used to draw Manhattan scatterplot, and the MultiExperiment Viewer (MeV) was applied to perform microarray clustering-analysis. Methylation involving gene promoters was defined if it occurred at regions of TSS1500 (upstream), TSS200 (upstream), 5′UTR and the first exon. Methylation involving the CpG island structures was defined as to occur at either CpG island per se or the N-Shore, S-Shore, N-Shelve and S-Shelve regions, with Shores occupying 0–2 kb from CpG island and Shelves covering 2–4 kb from the CpG island. Differential methylation genes were selected using the Database for Annotation, Visualization and Integrated Discovery (DAVID) bioinformatics database (http://david.abcc.ncifcrf.gov/home.jsp/). Differentially methylated genes were selected based on a P < 1.03e-07 for multi-factorial statistical comparison. Differential gene pathways were determined using the gene ontology (GO) (http://geneontology.org/) and Kyoto Encyclopedia of Genes and Genomes (KEGG) (http://www.genome.jp/kegg/) databases. Means of designated comparing groups were statistically analyzed with Student-*t* test (e.g., age comparison) or nonparametric test with Bonferroni correction (SPSS21.0), with the minimal significant level of difference set at P < 0.05. Correlation analyses and curve fitting were performed using the Prism 4 program (Prism GraphPad, San Diego, CA). Figure panels were assembled with Photoshop 7.1.

## Additional Information

**How to cite this article:** Long, H.-Y. *et al*. Blood DNA methylation pattern is altered in mesial temporal lobe epilepsy. *Sci. Rep.* **7**, 43810; doi: 10.1038/srep43810 (2017).

**Publisher's note:** Springer Nature remains neutral with regard to jurisdictional claims in published maps and institutional affiliations.

## Supplementary Material

Supplementary Information

## Figures and Tables

**Table 1:** Top ten hyper- and hypo- methylated genes in whole blood genome in MTLE.

	Target ID	Gene ID	Gene name	delta β	P value	CHR	Location	SNP
**Top ten hypermethylated genes**	cg01813209	ZNF638	zinc finger protein 638	0.05576	1.71E-08	2	Body	
	cg17461336	CYP3A43	cytochrome P450, family 3, subfamily A, polypeptide 43	0.05827	1.82E-08	7	Body	
	cg01123250	UNC80	unc-80 homolog	0.05829	6.63E-08	2	Body	rs75820072*
	cg25783241	AIRE	autoimmune regulator	0.06315	1.01E-09	21	Body	rs75243213^#^
	cg27133230	RBPJL	recombination signal binding protein for immunoglobulin kappa J region-like	0.06326	1.90E-08	20	Body	
	cg07532354	SCGB1D1	secretoglobin, family 1D, member 1	0.06374	3.22E-10	11	1st Exon	
	cg02711724	FAM49B	family with sequence similarity 49, member B	0.06567	2.45E-08	8	Body	
	cg27323430	FLJ44606	chromosome 5 open reading frame 63	0.06957	3.45E-10	5	Body	
	cg26094004	PYY	peptide YY	0.08969	1.78E-11	17	5′UTR	rs78314058*
	cg11792281	NLK	nemo-like kinase	0.09477	1.04E-08	17	Body	
**Top ten hypomethylated genes**	cg11582617	SERAC1	serine active site containing 1	−0.07829	4.66E-13	6	1st Exon	
	cg04908625	ADCY5	adenylate cyclase 5	−0.07580	2.13E-08	3	1st Exon	
	cg03782202	HOXD11	homeobox D11	−0.06426	1.16E-09	2	TSS1500	
	cg06852575	ZNF257	zinc finger protein 257	−0.05809	2.71E-08	19	Body	
	cg24200083	CLTCL1	clathrin, heavy chain-like 1	−0.05781	2.70E-08	22	TSS200	
	cg02440177	ZNF702P	zinc finger protein 702, pseudogene	−0.05330	9.05E-08	19	Body	
	cg15617548	GORASP2	golgi reassembly stacking protein 2	−0.05154	1.37E-09	2	5′UTR	
	cg25291941	POP1	POP1 homolog, ribonuclease P/MRP subunit	−0.04439	2.35E-08	8	TSS1500	
	cg25876975	TSPAN3	tetraspanin 3	−0.04368	5.62E-08	15	Body	
	cg20811730	PRPF3	pre-mRNA processing factor 3	−0.04317	8.26E-08	1	TSS200	

CHR: chromosome; SNP: single nucleotide polymorphism, *within probes > 10 bp and ^#^< 10 bp from query sites.

**Table 2:** Genomic networks with altered methylation in MTLE.

Pathway list	Pathway term	# DMS involved	% to total DMS	Genes	P Value	Fold Enriched
#1	hsa00591:Linoleic acid metabolism	4	2.5974	CYP3A43, CYP3A4, ALOX15, CYP2C9	0.0017	16.1429
#2	hsa00983:Drug metabolism	4	2.5974	CYP3A43, CYP3A4, NAT2, UGT2A3	0.0059	10.5116
#3	hsa00830:Retinol metabolism	4	2.5974	CYP3A43, CYP3A4, CYP2C9, UGT2A3	0.0110	8.3704
#4	hsa00980:Metabolism of xenobiotics by cytochrome P450	4	2.5974	CYP3A43, CYP3A4, CYP2C9, UGT2A3	0.0147	7.5333
#5	hsa00982:Drug metabolism	4	2.5974	CYP3A43, CYP3A4, CYP2C9, UGT2A3	0.0160	7.2903
#6	hsa00140:Steroid hormone biosynthesis	3	1.9481	CYP3A43, CYP3A4, UGT2A3	0.0596	7.3696

**Table 3:** Demographic, clinical and brain imaging profile of the subjects enrolled.

		MTLE (n = 30)	Control (n = 30)
Male (n = 18)	Age (mean ± SD)*	25.28 ± 11.75	28.17 ± 13.96
Female (n = 12)	Age (mean ± SD)*	33.33 ± 14.28	35.83 ± 11.19
Seizure type	dyscognitive seizures	1 (3.33%)	
	Automation	7 (23.33%)	
	generalized tonic-clonic seizures (GTCS)	7 (23.33%)	
	GTCS + dyscognitive seizures	1 (3.33%)	
	GTCS + automation	9 (30.00%)	
	dyscognitive seizures + automation	4 (13.33%)	
	GTCS + dyscognitive seizures + automation	1 (3.33%)	
Aura	None	12 (40.00%)	
	Affective	1 (3.33%)	
	Epigastric	7 (23.33%)	
	Cephalic	4 (13.33%)	
	Palpitations	3 (10.00%)	
	Visual	2 (6.67%)	
Inter-ictal EEG	Left temporal	10 (33.33%)	
	Right temporal	11 (36.67%)	
	Bilateral temporal	9 (30.00%)	
MRI	Normal	14 (46.67%)	
	Hippocampal sclerosis	9 (30.00%)	
	Encephalomal acia foci	1 (3.33%)	
	Lacunar infarction	4 (13.33%)	
	Encephalomal atrophy	2 (6.67%)	
Seizure frequency	>Once per week	8 (26.67%)	
	Once per week to month	6 (20.00%)	
	Once per month to 6 months	10 (33.33%)	
	<once per year	6 (20.00%)	
Disease duration	<10 years	10 (33.33%)	
	≥10 years	20 (66.67%)	
Age of first onset	Within 1 year	1 (3.33%)	
	1–12 years	9 (30.00%)	
	>12 years	20 (66.67%)	

*P > 0.05 between the patient and control groups by Student-*t* test.
