# Supplementary material for: Blood DNA methylation pattern is altered in mesial temporal lobe epilepsy
Source: Sci Rep. 2017 Mar 9;7:43810. doi: 10.1038/srep43810 (PMC5343463; doi:10.1038/srep43810)
Supplement: Supplementary Information [file srep43810-s1.pdf]

Supplementary information

SREP-15-32874C

Blood DNA methylation pattern is altered in mesial temporal lobe epilepsy

Hong-Yu Long, Li Feng, Jin Kang, Zhao-Hui Luo, Wen-Biao Xiao, Li-Li Long,
Xiao-Xin Yan, Luo Zhou & Bo Xiao

Supplementary Figure 1-4

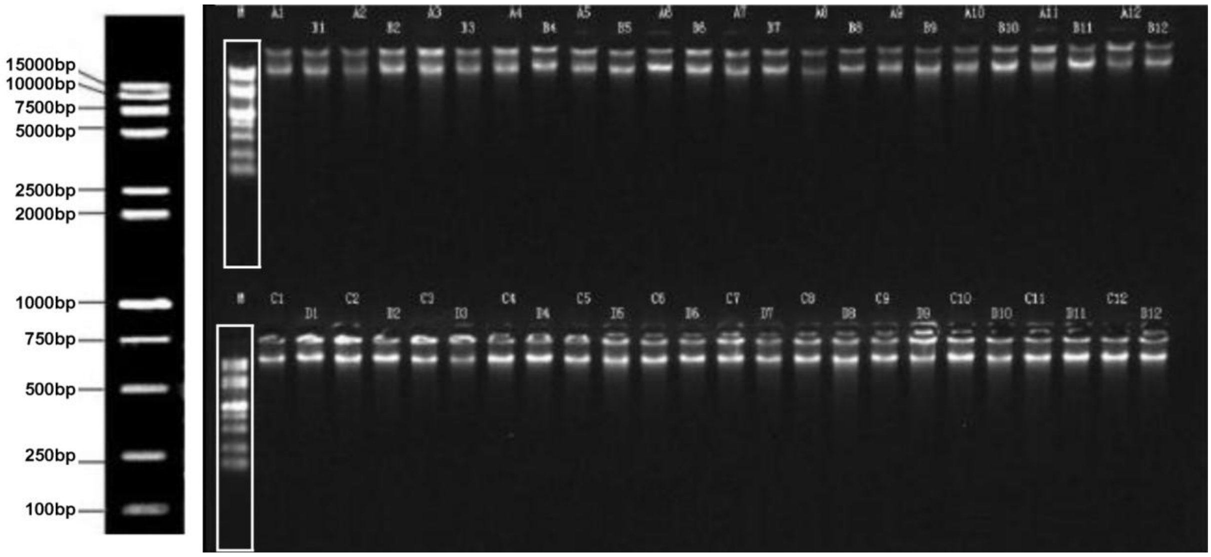

Supplementary Figure 1. Representative image of ethidium bromide stained agarose gel illustrating the distribution pattern of purified DNA from blood samples of patients and controls in electrophoresis. The bulk of DNA fragments appear as two distinct bands migrated at the locations corresponding to molecular sizes apparently greater than 15000 base-pairs (bp).

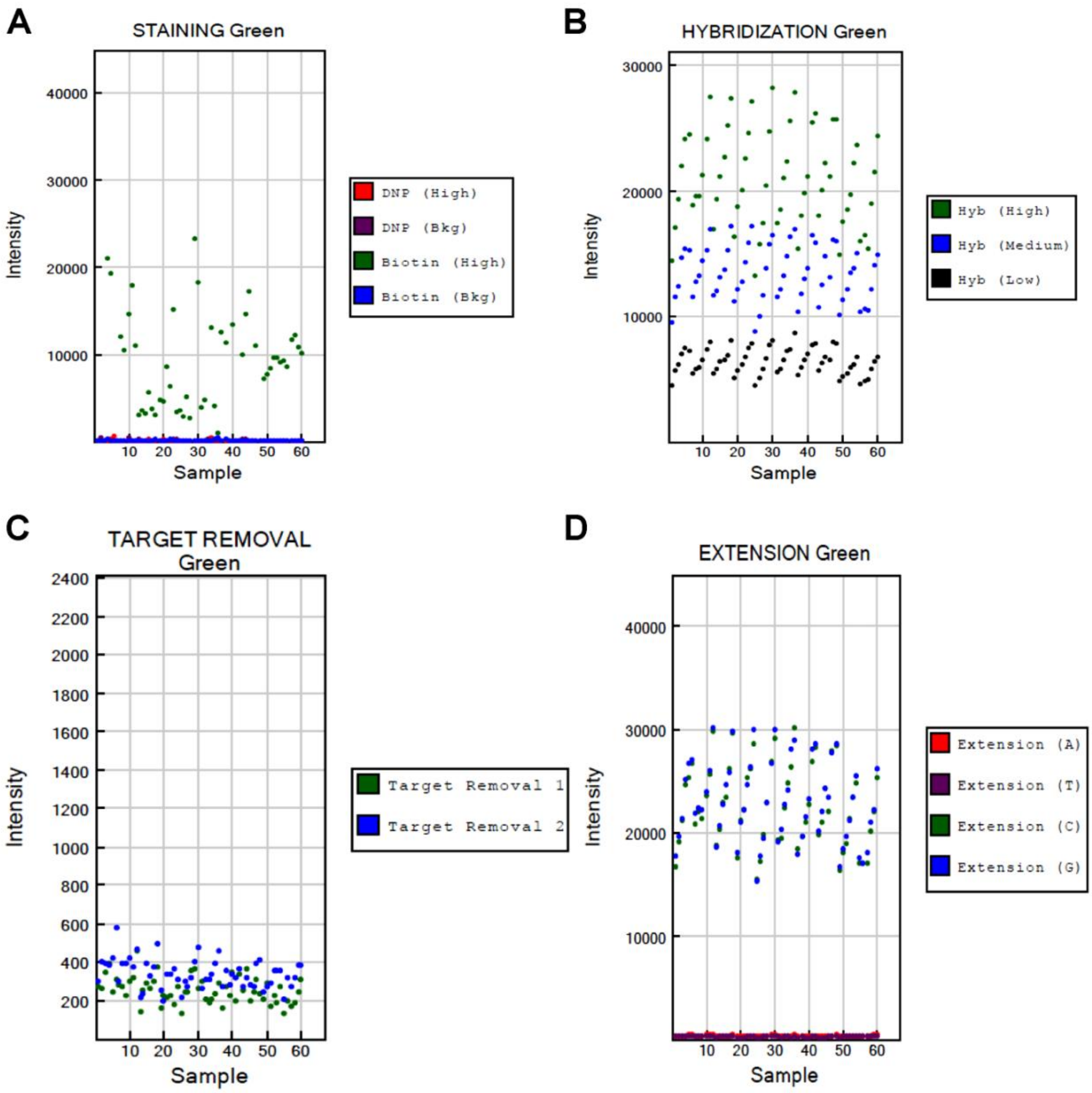

Supplementary Figure 2. Spot graphs illustrating the results of sample-independent assay controls. Panels (A-D) represent intensity of fluorescent signal in the Staining, Hybridization, Target Removal and Extension controls, as indicated.

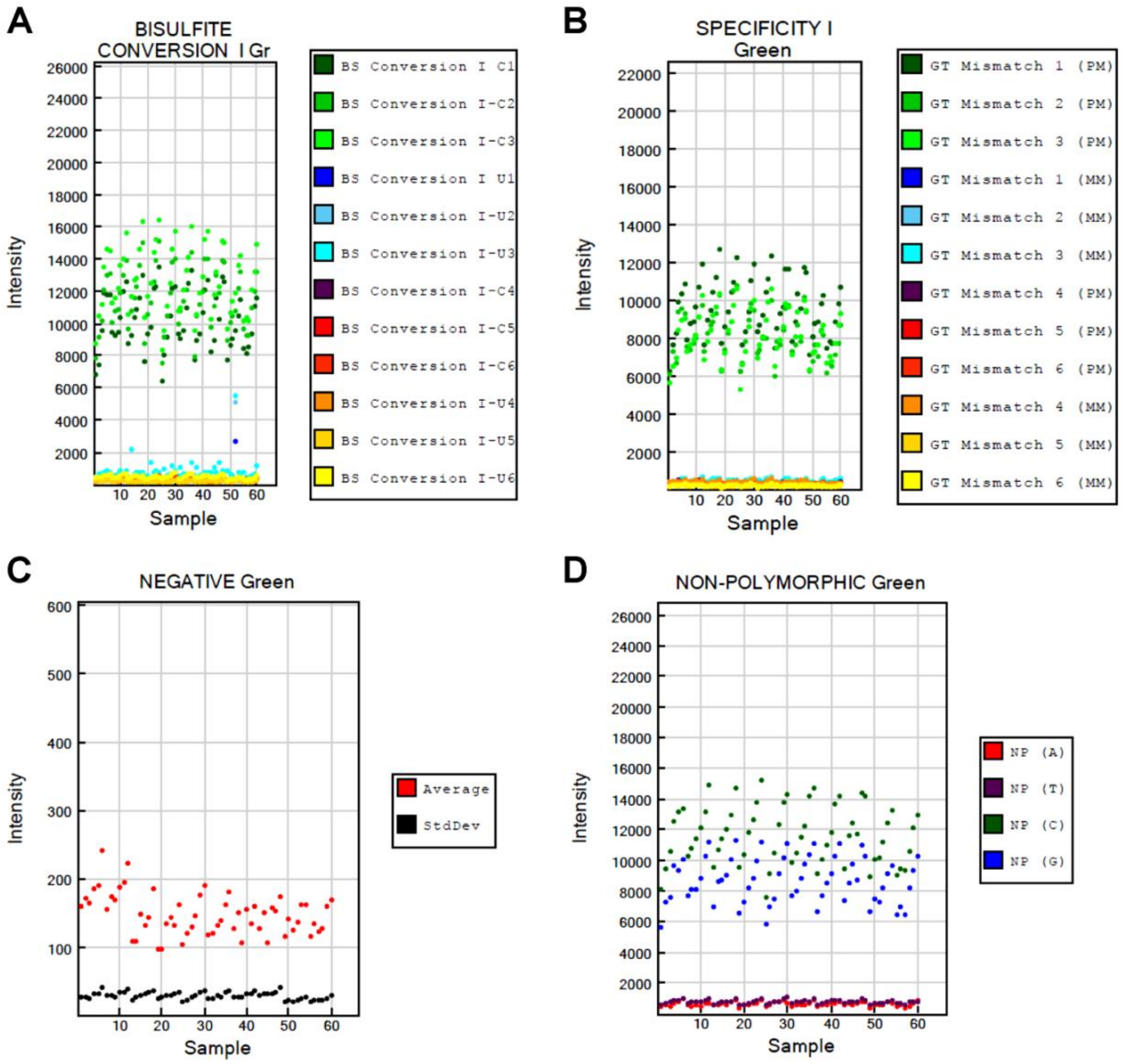

Supplementary Figure 3. Spot graphs illustrating the results of sample-dependent assay controls. Results from the Infmium-I assay controls are shown in (A, B) . Results from the In Infmium-II assay controls are shown in (C, D).

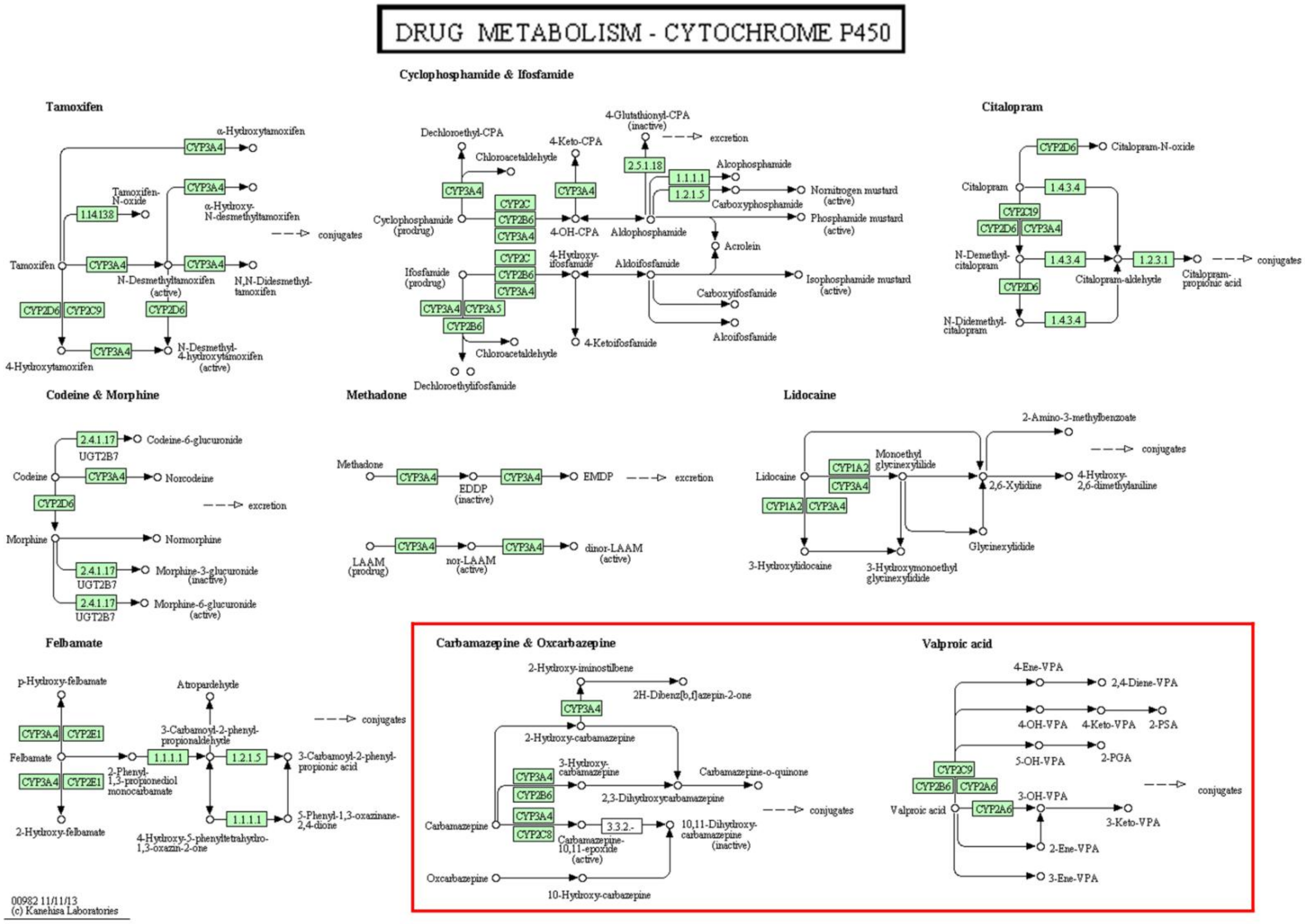

Supplementary Figure 4. Bioinformatics profiling of the cytochrome P450 system in relative to drug metabolism. The enzyme system corresponds to the hsa00980 pathway according to the KEGG database/software (http://www.genome.jp/kegg/, obtained with permission, ref. #16835). Enzymes coded by the corresponding genes participate in the metabolism of many drugs including Tamoxifen, Cyclophosphamide, Isosfamide, Citalopram, Codeine, Morphine, Methadone, Lidocaine, Felbamate, Carbamazepine and Oxcarbazepine.

<u>Supplementary Tables 1-7</u>

Supplementary Table 1: Differentially methylated genes in blood whole-genome of MTLE relative to control subjects (listed alphabetically)

Gene ID	Gene Name
	**Hypermethylated genes (n=87)**
**ACTN4**	ADAM metallopeptidase with thrombospondin type 1 motif, 20
**ADAMTS20**	ADP-ribosyltransferase 3
**ADAMTS7**	AF4/FMR2 family, member 3
**AFF3**	ATPase, Na+/K+ transporting, alpha 1 polypeptide
**AIRE**	BAH domain and coiled-coil containing 1
**ANKRD11**	BAI1-associated protein 2
**ARSI**	BEN domain containing 3
**ART3**	DENN/MADD domain containing 1C
**ATP1A1**	E4F transcription factor 1
**BAHCC1**	FCH domain only 2
**BAI1**	GABA(A) receptors associated protein like 3 (pseudogene); GABA(A) receptor-associated protein like 1
**BAIAP2**	KIAA1688 protein
**BEND3**	MCF.2 cell line derived transforming sequence-like
**BRD1**	MHC class I polypeptide-related sequence A
**BRD4**	N-acetyltransferase 2 (arylamine N-acetyltransferase)
**C1orf203**	NADH dehydrogenase (ubiquinone) 1 beta subcomplex, 6, 17kDa
**C20orf152**	NHS-like 1
**CDK10**	NOP56 ribonucleoprotein homolog (yeast)
**CLCA4**	RNA binding motif, single stranded interacting protein 1
**CLCN6**	SET domain containing 1B
**COL11A2**	WD repeat domain 46
**CSPG4**	actinin, alpha 4
**CXCL11**	ankyrin repeat domain 11; hypothetical protein LOC100128265
**CXCR1**	arylsulfatase family, member I
**CYP2C9**	autoimmune regulator
**CYP3A4**	brain-specific angiogenesis inhibitor 1
**CYP3A43**	bromodomain containing 1
**CYTH1**	bromodomain containing 4
**DENND1C**	chemokine (C-X-C motif) ligand 11
**DES**	chloride channel 6
**DNAH17**	chloride channel accessory 4
**E4F1**	chondroitin sulfate proteoglycan 4

**FAM122A**	chromosome 1 open reading frame 203
**FAM49B**	chromosome 20 open reading frame 152
**FBRSL1**	collagen, type XI, alpha 2
**FCHO2**	cyclin-dependent kinase 10
**FLJ44606**	cytochrome P450, family 2, subfamily C, polypeptide 9
**FSD1L**	cytochrome P450, family 3, subfamily A, polypeptide 4
**FZD5**	cytochrome P450, family 3, subfamily A, polypeptide 43
**GABARAPL3**	cytohesin 1
**GABRB1**	desmin
**HIST3H3**	dynein, axonemal, heavy chain 17
**HYLS1**	family with sequence similarity 122A
**KIAA1688**	family with sequence similarity 49, member B
**KLK2**	fibronectin type III and SPRY domain containing 1-like
**LOC100216001**	fibrosin-like 1
**LRP5**	frizzled homolog 5 (Drosophila)
**MAEL**	gamma-aminobutyric acid (GABA) A receptor, beta 1
**MAPT**	histone cluster 3, H3
**MCF2L**	hydrolethalus syndrome 1
**MICA**	hypothetical LOC100216001
**MIR526A2**	hypothetical protein LOC401207
**NAT2**	interleukin 8 receptor, alpha
**NDUFB6**	kallikrein-related peptidase 2
**NHSL1**	low density lipoprotein receptor-related protein 5
**NLK**	maelstrom homolog (Drosophila)
**NME2P1**	microRNA 526a-2
**NOP56**	microtubule-associated protein tau
**NPR1**	natriuretic peptide receptor A/guanylate cyclase A (atrionatriuretic peptide receptor A)
**NRBP2**	nemo-like kinase
**PFDN6**	non-metastatic cells 2, protein (NM23B) expressed in, pseudogene 1
**PIP5K1B**	nuclear receptor binding protein 2
**PKP4**	pancreatic polypeptide 2
**PLXND1**	peptide YY
**PPCDC**	phosphatidylinositol-4-phosphate 5-kinase, type I, beta
**PPY2**	phosphopantothenoylcysteine decarboxylase
**PYY**	plakophilin 4
**RBMS1**	plexin D1
**RBPJL**	prefoldin subunit 6
**RHPN1**	recombination signal binding protein for immunoglobulin kappa J region-like
**SCGB1D1**	rhophilin, Rho GTPase binding protein 1
**SETD1B**	secretoglobin, family 1D, member 1
**SLC34A2**	similar to hCG1991431; similar to COMPase; ADAM metallopeptidase with thrombospondin type 1 motif, 7
**SLC6A17**	small nucleolar RNA, C/D box 57
**SLC7A14**	solute carrier family 34 (sodium phosphate), member 2

**SNORD57**	solute carrier family 6, member 17
**SPDYE4**	solute carrier family 7 (cationic amino acid transporter, y+ system), member 14
**ST20**	speedy homolog E4 (Xenopus laevis)
**SYT16**	suppressor of tumorigenicity 20
**TGM1**	synaptotagmin XVI
**THSD7B**	tetratricopeptide repeat domain 7B
**TRIM65**	thrombospondin, type I, domain containing 7B
**TTC7B**	transglutaminase 1 (K polypeptide epidermal type I, protein-glutamine-gamma-glutamyltransferase)
**TTLL2**	tripartite motif-containing 65
**WDR46**	tubulin tyrosine ligase-like family, member 2
**ZBTB45**	zinc finger and BTB domain containing 45
**ZNF638**	zinc finger protein 638
**Hypomethylated genes (n=43)**	
**ADCY5**	AFG3 ATPase family gene 3-like 2 (yeast)
**AFG3L2**	HLA-B associated transcript 2
**AK3**	PRP3 pre-mRNA processing factor 3 homolog (S. cerevisiae)
**ALOX15**	SH3 domain containing 20 pseudogene
**BACE1**	Sin3A-associated protein, 30kDa
**BAHD1**	U2 small nuclear RNA auxiliary factor 1
**BAT2**	adenylate cyclase 5
**BLVRA**	adenylate kinase 3
**C1GALT1**	antigen p97 (melanoma associated) identified by monoclonal antibodies 133.2 and 96.5
**C1QL1**	arachidonate 15-lipoxygenase
**C9orf171**	beta-site APP-cleaving enzyme 1
**CCDC48**	biliverdin reductase A
**CLTCL1**	bromo adjacent homology domain containing 1
**DLL3**	chromosome 9 open reading frame 171
**FAHD1**	clathrin, heavy chain-like 1
**FAM91A1**	coiled-coil domain containing 48
**GNG7**	complement component 1, q subcomponent-like 1
**GORASP2**	core 1 synthase, glycoprotein-N-acetylgalactosamine 3-beta-galactosyltransferase, 1
**GTF2H5**	delta-like 3 (Drosophila)
**HAGH**	family with sequence similarity 91, member A1
**HNRNPF**	fumarylacetoacetate hydrolase domain containing 1
**HOXB2**	general transcription factor IIH, polypeptide 5
**HOXD11**	golgi reassembly stacking protein 2, 55kDa
**HRSP12**	guanine nucleotide binding protein (G protein), gamma 7
**IGF2BP1**	heat-responsive protein 12
**KY**	heterogeneous nuclear ribonucleoprotein F
**LOC440461**	homeobox B2
**MFI2**	homeobox D11
**NUCKS1**	hydroxyacylglutathione hydrolase
**POP1**	insulin-like growth factor 2 mRNA binding protein 1

**PPP2CA**	kyphoscoliosis peptidase
**PRPF3**	nuclear casein kinase and cyclin-dependent kinase substrate 1
**PURG**	processing of precursor 1, ribonuclease P/MRP subunit (S. cerevisiae)
**SAP30**	protein phosphatase 2 (formerly 2A), catalytic subunit, alpha isoform
**TAPT1**	purine-rich element binding protein G
**TLE3**	similar to Werner syndrome protein; Werner syndrome, RecQ helicase-like
**TMED10**	tetratricopeptide repeat domain 7B
**TMX1**	thioredoxin-related transmembrane protein 1
**TTC7B**	transducin-like enhancer of split 3 (E(sp1) homolog, Drosophila)
**U2AF1**	transmembrane anterior posterior transformation 1
**WRN**	transmembrane emp24-like trafficking protein 10 (yeast)
**YWHAQ**	tyrosine 3-monooxygenase/tryptophan 5-monooxygenase activation protein, theta polypeptide
**ZNF815**	zinc finger protein 815

Supplementary Table 2: Relevance of differentially methylated DNA sites to cellular component (CC) genes

Pathway list	Pathway term	# DMS involved	% to total DMS	Genes	P Value	Fold Enriched	FDR
#1	GO:0030529~ribonucleoprotein complex	8	5.1948	ACTN4, HNRNPF, U2AF1, POP1, MAEL, IGF2BP1, PRPF3, NOP56	0.0775	2.1350	P>0.05
#2	GO:0005792~microsome	5	3.2468	CYP3A43, CYP3A4, CYP2C9, TMED10, ATP1A1	0.0915	2.8996	P>0.05
#3	GO:0031981~nuclear lumen	16	10.3896	BRD1, ACTN4, NLK, SETD1B, IGF2BP1, PRPF3, WRN, ZNF638, SAP30, HNRNPF, U2AF1, POP1, BRD4, NOP56, E4F1, HIST3H3	0.0942	1.5166	P>0.05
#4	GO:0042598~vesicular fraction	5	3.2468	CYP3A43, CYP3A4, CYP2C9, TMED10, ATP1A1	0.0992	2.8164	P>0.05

Supplementary Table 3: Relevance of differentially methylated DNA sites to biological process (BP) genes

Pathway list	Pathway term	# DMS involved	% to total DMS	Genes	P Value	Fold Enriched	FDR
#1	GO:0040008~regulation of growth	8	5.1948	TMX1, MAPT, PPP2CA, MAEL, NPR1, WRN, E4F1, AFG3L2	0.0101	3.3060	P>0.05
#2	GO:0001501~skeletal system development	7	4.5455	TAPT1, ALOX15, HOXB2, ANKRD11, DLL3, COL11A2, HOXD11	0.0248	3.0922	P>0.05
#3	GO:0048705~skeletal system morphogenesis	4	2.5974	HOXB2, ANKRD11, COL11A2, HOXD11	0.0442	5.0327	P>0.05
#4	GO:0016055~Wnt receptor signaling pathway	4	2.5974	NLK, TLE3, FZD5, LRP5	0.0670	4.2381	P>0.05
#5	GO:0009952~anterior/posterior pattern formation	4	2.5974	HOXB2, DLL3, LRP5, HOXD11	0.0756	4.0262	P>0.05
#6	GO:0009123~nucleoside monophosphate metabolic process	3	1.9481	ADCY5, AK3, NPR1	0.0784	6.4053	P>0.05
#7	GO:0010720~positive regulation of cell development	3	1.9481	MAPT, DLL3, HOXD11	0.0846	6.1268	P>0.05
#8	GO:0006164~purine nucleotide biosynthetic process	4	2.5974	ADCY5, AK3, NPR1, ATP1A1	0.0860	3.8086	P>0.05
#9	GO:0006396~RNA processing	8	5.1948	PPP2CA, HNRNPF, U2AF1, POP1, PRPF3, ZNF638, NOP56, RBMS1	0.0898	2.0609	P>0.05

Supplementary Table 4: Relevance of differentially methylated DNA sites to molecular function (MF) genes

Pathway list	Pathway term	# DMS involved	% to total DMS	Genes	P Value	Fold Enriched	FDR
#1	GO:0043168~anion binding	5	3.2468	CLCA4, GABRB1, NPR1, CLCN6, SLC34A2	0.0055	6.9861	P>0.05
#2	GO:0043167~ion binding	44	28.5714	CYP3A4, OCM, ADCY5, GABRB1, ARSI, ZNF638, ZKSCAN3, CYP3A43, FAHD1, TRIM65, ZNF323, GALNTL5, ZNF429, PPP2CA, TGM1, U2AF1, CALN1, E4F1, C1GALT1, BRD1, KLF7, HPCAL1, CLCA4, ACTN4, CYP2C9, ADAMTS20, NLK, MGAT4C, MFI2, NPR1, ZNF8, ATP1A1, ZBTB45, AFG3L2, SLC34A2, ADAMTS7, HAGH, BLVRA, ALOX15, VSNL1, AIRE, CLCN6, ZNF702P, ZNF257	0.0192	1.3336	P>0.05
#3	GO:0031404~chloride ion binding	4	2.5974	CLCA4, GABRB1, NPR1, CLCN6	0.0215	6.6776	P>0.05
#4	GO:0016712~oxidoreductase activity, acting on paired donors, with incorporation or reduction of molecular oxygen, reduced flavin or flavoprotein as one donor, and incorporation of one atom of oxygen	3	1.9481	CYP3A43, CYP3A4, CYP2C9	0.0222	12.8545	P>0.05

#5	GO:0004886~retinoid-X receptor activity	2	1.2987	PFDN6, COL11A2	0.0672	28.5655	P>0.05
#6	GO:0046872~metal ion binding	40	25.9740	CYP3A4, OCM, ADCY5, ARSI, ZNF638, ZKSCAN3, CYP3A43, FAHD1, TRIM65, ZNF323, GALNTL5, ZNF429, PPP2CA, TGM1, U2AF1, CALN1, E4F1, C1GALT1, BRD1, KLF7, HPCAL1, ACTN4, CYP2C9, ADAMTS20, NLK, MGAT4C, MFI2, ZNF8, ATP1A1, ZBTB45, AFG3L2, SLC34A2, BLVRA, HAGH, ADAMTS7, ALOX15, VSNL1, AIRE, ZNF702P, ZNF257	0.0788	1.2420	P>0.05
#7	GO:0030375~thyroid hormone receptor coactivator activity	2	1.2987	PFDN6, COL11A2	0.0816	23.3717	P>0.05
#8	GO:0010861~thyroid hormone receptor activator activity	2	1.2987	PFDN6, COL11A2	0.0816	23.3717	P>0.05
#9	GO:0003708~retinoic acid receptor activity	2	1.2987	PFDN6, COL11A2	0.0886	21.4241	P>0.05
#10	GO:0043169~cation binding	40	25.9740	CYP3A4, OCM, ADCY5, ARSI, ZNF638, ZKSCAN3, CYP3A43, FAHD1, TRIM65, ZNF323, GALNTL5, ZNF429, PPP2CA, TGM1, U2AF1, CALN1, E4F1, C1GALT1, BRD1, KLF7, HPCAL1, ACTN4, CYP2C9, ADAMTS20, NLK, MGAT4C, MFI2, ZNF8, ATP1A1, ZBTB45, AFG3L2, SLC34A2, BLVRA, HAGH, ADAMTS7, ALOX15, VSNL1, AIRE, ZNF702P, ZNF257	0.0890	1.2304	P>0.05
#11	GO:0005506~iron ion binding	6	3.8961	CYP3A43, CYP3A4, ALOX15, CYP2C9, PPP2CA, MFI2	0.0892	2.5041	P>0.05

Supplementary Table 5: Blood DNA methylation analysis in the MTLE group in relevance to disease duration

	Target ID	Gene ID	Gene name	delta β	P (*t*-test)	CHR	Location	SNP
**Top 10 different sites (<10 years vs. ≥10 years)**	cg02621636	MS4A14;MS4A7	membrane spanning 4-domains A14,membrane spanning 4-domains A7	0.02199	8.85E-06	11	TSS1500;3'UTR	
	cg01194712	C14orf159	chromosome 14 open reading frame 159	-0.01077	1.52E-05	14	Body	
	cg25401754	FBXO5	F-box protein 5	-0.00691	1.78E-05	6	5'UTR;TSS200	
	cg24448167	PRDM15	PR/SET domain 15	-0.02260	1.91E-05	21	Body; Body	
	cg06093993	TAAR2	trace amine associated receptor 2	0.02896	2.17E-05	6	TSS200	
	cg04626105	RAB9A	RAB9A, member RAS oncogene family	0.04299	2.24E-05	X	Body	
	cg21569398	ADAMTS12	ADAM metallopeptidase with thrombospondin type 1 motif 12	-0.05868	3.47E-05	5	Body	
	cg27000496	SLC45A4	solute carrier family 45 member 4	-0.00863	3.71E-05	8	Body	
	cg00164546	LDLRAP1	low density lipoprotein receptor adaptor protein 1	-0.03060	4.10E-05	1	Body	
	cg11305009			-0.01747	4.22E-05	7		
**Top 10 hypermethylated (≥10 years)**	cg02621636	MS4A14;MS4A7	membrane spanning 4-domains A14； membrane spanning 4-domains A7	0.02199	8.85E-06	11	TSS1500;3'UTR	
	cg06093993	TAAR2	trace amine associated receptor 2	0.02896	2.17E-05	6	TSS200	
	cg04626105	RAB9A	RAB9A, member RAS oncogene family	0.04299	2.24E-05	X	Body	
	cg26195168			0.03004	5.70E-05	7		
	cg19556599	BRD9	bromodomain containing 9	0.01660	7.10E-05	5	Body	
	cg01790920	IRX1	iroquois homeobox 1	0.12606	7.89E-05	5	TSS1500	rs828336
	cg00451039	LEPRE1;C1orf50	prolyl 3-hydroxylase 1； chromosome 1 open reading frame 50	0.00699	8.88E-05	1	TSS1500;Body	
	cg06821789	LYRM5	electron transfer flavoprotein regulatory factor 1	0.01805	9.56E-05	12	5'UTR	rs74073341*
	cg25936770	CADPS2	calcium dependent secretion activator 2	0.02479	1.13E-04	7	Body	
	cg07161146	PRKAG2	protein kinase AMP-activated non-catalytic subunit gamma 2	0.02320	1.16E-04	7	Body	
**Top 10 hypomethylated (≥10 years)**	cg01194712	C14orf159	chromosome 14 open reading frame 159	-0.01077	1.52E-05	14	Body	
	cg25401754	FBXO5	F-box protein 5	-0.00691	1.78E-05	6	5'UTR;TSS200	
	cg24448167	PRDM15	PR/SET domain 15	-0.02260	1.91E-05	21	Body	
	cg21569398	ADAMTS12	ADAM metallopeptidase with thrombospondin type 1 motif 12	-0.05868	3.47E-05	5	Body	
	cg27000496	SLC45A4	solute carrier family 45 member 4	-0.00863	3.71E-05	8	Body	
	cg00164546	LDLRAP1	low density lipoprotein receptor adaptor protein 1	-0.03060	4.10E-05	1	Body	
	cg11305009			-0.01747	4.22E-05	7		
	cg08357850	MLKL	mixed lineage kinase domain like	-0.01743	5.51E-05	16	TSS200	
	cg20312087	MGC70857	chromosome 8 open reading frame 82	-0.01059	6.48E-05	8	1stExon	
	cg20447301	RBM33	RNA binding motif protein 33	-0.00926	7.41E-05	7	3'UTR	

Supplementary Table 6: Blood DNA methylation analysis in the MTLE group in relevance to anti-epileptics resistance

	Target ID	Gene ID	Gene name	delta β	P (*t*-test)	CHR	Location	SNP
Top 10 different (drug resistant vs. sensitive)	cg25348323			0.04717	7.04E-07	7		
	cg26917132	FBXO31;MAP1LC3B	F-box protein 31； microtubule associated protein 1 light chain 3 beta	-0.00629	3.17E-06	16	TSS200	
	cg25045882			0.01697	5.30E-06	11		rs1789803*
	cg23753807	ABR	active BCR-related	0.01349	8.45E-06	17	1stExon;5'UTR	rs72816208*
	cg03408024			0.04707	1.48E-05	4		
	cg05624199			0.04038	2.28E-05	19		
	cg12683641	KLK14	kallikrein related peptidase 14	0.03601	2.32E-05	19	5'UTR	
	cg04307702	ALLC	allantoicase	0.01987	2.53E-05	2	5'UTR	
	cg01282661	MAGIX	MAGI family member, X-linked	0.03392	3.44E-05	X	TSS1500	
	cg24485927			0.03803	3.56E-05	2		
Top 10 hypermethylated (drug resistant)	cg25348323			0.04717	7.04E-07	7		
	cg25045882			0.01697	5.30E-06	11		rs1789803*
	cg23753807	ABR	active BCR-related	0.01349	8.45E-06	17	1stExon;5'UTR	rs72816208*
	cg03408024			0.04707	1.48E-05	4		
	cg05624199			0.04038	2.28E-05	19		
	cg12683641	KLK14	kallikrein related peptidase 14	0.03601	2.32E-05	19	5'UTR	
	cg04307702	ALLC	allantoicase	0.01987	2.53E-05	2	5'UTR	
	cg01282661	MAGIX	MAGI family member, X-linked	0.03392	3.44E-05	X	TSS1500	
	cg24485927			0.03803	3.56E-05	2		
	cg08637446			0.00853	3.60E-05	11		
Top 10 hypomethylated (drug resistant)	cg26917132	FBXO31;MAP1LC3B	F-box protein 31； microtubule associated protein 1 light chain 3 beta	-0.00629	3.17E-06	16	TSS200	
	cg08873805	ZSCAN18	zinc finger and SCAN domain containing 18	-0.01819	6.07E-05	19	TSS200	rs8108978
	cg04678989	ACTN4	actinin alpha 4	-0.01376	7.29E-05	19		
	cg10191240	WISP1	WNT1 inducible signaling pathway protein 1	-0.04334	8.81E-05	8		
	cg07772145	TPRA1	transmembrane protein adipocyte associated 1	-0.00418	9.73E-05	3		
	cg05800928	HSPB1	heat shock protein family B (small) member 1	-0.01181	1.18E-04	7		rs7457776*
	cg05322837			-0.04472	1.46E-04	17		
	cg13971154	SMO	smoothened, frizzled class receptor	-0.00892	1.54E-04	7		
	cg17707870	LHFP	lipoma HMGIC fusion partner	-0.37111	1.61E-04	13		
	cg25649000	PTPRT	protein tyrosine phosphatase, receptor type T	-0.02090	1.80E-04	20		rs75204617

Supplementary Table 7: Blood DNA methylation analysis in the MTLE group in relevance to MRI abnormality of hippocampal sclerosis (HS)

	Target ID	Gene ID	Gene name	delta β	P (*t*-test)	CHR	Location	SNP
Top 10 different sites (HS vs. normal in MRI)	cg01979298			-0.61089	2.34E-05	17		rs56394376
	cg04663690	PCNT	pericentrin	-0.02203	3.92E-05	21	Body	
	cg06670612	CEACAM21	carcinoembryonic antigen related cell adhesion molecule 21	-0.00751	3.97E-05	19	TSS200	
	cg15645804	PCGF3	polycomb group ring finger 3	-0.02388	5.19E-05	4	Body	rs73222807
	cg22673380	SLC12A7	solute carrier family 12 member 7	0.00690	5.25E-05	5	Body	
	cg14381040			-0.02870	6.18E-05	6		
	cg23635789	SRC	SRC proto-oncogene, non-receptor tyrosine kinase	0.04239	6.29E-05	20	Body	
	cg23254302	PLIN5	perilipin 5	0.05849	6.92E-05	19	Body	
	cg18176509			-0.01760	7.18E-05	1		rs12122113*
	cg25515481	BAT2	proline rich coiled-coil 2A	-0.02744	8.21E-05	6	Body	
Top 10 hypermethylated (with MRI sign of HS)	cg22673380	SLC12A7	solute carrier family 12 member 7	0.00690	5.25E-05	5	Body	
	cg23635789	SRC	SRC proto-oncogene, non-receptor tyrosine kinase	0.04239	6.29E-05	20	Body	
	cg23254302	PLIN5	perilipin 5	0.05849	6.92E-05	19	Body	
	cg21002957	RASAL3	RAS protein activator like 3	0.02617	1.00E-04	19	Body	
	cg00133147	PFDN6;WDR46	prefoldin subunit 6， WD repeat domain 46	0.01357	1.06E-04	6	TSS1500;Body	
	cg18354686	UBN1;GLYR1	ubinuclein 1;glyoxylate reductase 1 homolog	0.00759	1.13E-04	16	TSS1500;TSS200	
	cg05444785	TRIM49	tripartite motif containing 49	0.04713	1.25E-04	11	TSS1500	
	cg14378593	ACVR1B	activin A receptor type 1B	0.01043	1.36E-04	12	TSS1500	
	cg24479382			0.10861	1.43E-04	21		rs5844228*
	cg13374026			0.04135	1.43E-04	7		
Top 10 hypomethylated (with MRI sign of HS)	cg01979298			-0.61089	2.34E-05	17		rs56394376
	cg04663690	PCNT	pericentrin	-0.02203	3.92E-05	21	Body	
	cg06670612	CEACAM21	carcinoembryonic antigen related cell adhesion molecule 21	-0.00751	3.97E-05	19	TSS200	
	cg15645804	PCGF3	polycomb group ring finger 3	-0.02388	5.19E-05	4	Body	rs73222807
	cg14381040			-0.02870	6.18E-05	6		
	cg18176509			-0.01760	7.18E-05	1		rs12122113*
	cg25515481	BAT2	proline rich coiled-coil 2A	-0.02744	8.21E-05	6	Body	
	cg27031117	SPIRE2	spire type actin nucleation factor 2	-0.00729	8.66E-05	16	Body	
	cg17804302	NKD1	naked cuticle homolog 1	-0.01334	8.97E-05	16	TSS1500	
	cg22750483	TNFRSF11B	TNF receptor superfamily member 11b	-0.00954	9.25E-05	8	1stExon;5'UTR	
